# Non-concentric Reduction Due to Entrapped Loose Bone Piece During a Single-stage Bilateral Total Hip Arthroplasty

**DOI:** 10.7759/cureus.3780

**Published:** 2018-12-27

**Authors:** Mantu Jain, Samrat S Sahoo, Nabin K Sahu, Rajesh Rana, Sudarsan Behera

**Affiliations:** 1 Orthopaedics, All India Institute of Medical Sciences, Bhubaneswar, IND; 2 Orthopaedics, All India Institute of Medical Sciences, Bhubaneswar , IND

**Keywords:** total hip arthroplasty, non-concentric reduction, intra-articular osteophyte

## Abstract

Total hip arthroplasty (THA) is the treatment of choice for Grade IV avascular necrosis of the femoral head. Dislocation following THA, although rare, is a known complication. Common causes of unsuccessful reduction include interposition of soft tissue, component loosening, malalignment, and inadequate muscle relaxation following anaesthesia. Here, we encountered a rare complication during a single-stage bilateral THA that resulted in a non-concentric reduction on the left side. The pathology was a loose bone piece, which possibly was an osteophyte that was broken.

## Introduction

Total hip arthroplasty (THA) has gratifying results for patients with hip ailments [1]. However, the procedure is associated with certain complications [2]. The incidence of dislocation following primary THA is approximately 1-6% [3]. A non-concentric reduction in the immediate postoperative period must alert the treating surgeon to the presence of an intra-articular material. We encountered such a case in a bilateral, single-stage THA where the patient was operated on for Grade IV avascular necrosis of the femoral head; we observed a non-concentric reduction on the left side due to an entrapped osteophyte.

## Case presentation

**Figure 1:**
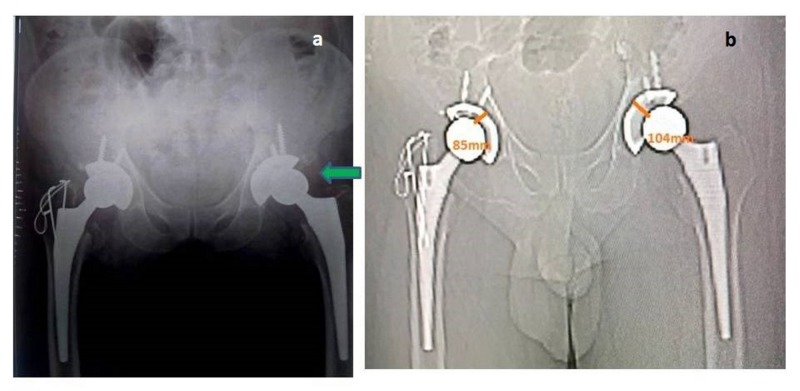
Imaging study. a) X-ray pelvis of the bilateral hip showing a concentric reduction on the right side and a non-concentric reduction on the left side. b) The scanogram depicts an increase in the distance from the center of the acetabular shell to that of the femoral head.

A 58-year-old man with Grade IV bilateral avascular necrosis underwent a single-stage THA (metal on polyethylene; Duraloc, Johnson & Johnson, USA). The left side was operated upon first through a modified Hardinge approach and, owing to greater trochanter partial avulsion, a tension band wiring was undertaken. On the right side, the THA was uneventful. The post-reduction stability and range of movements were checked and the wound was closed. A bedside X-ray showed a proud head and a non-centric reduction on the left THA (Figure 1a).

**Figure 2:**
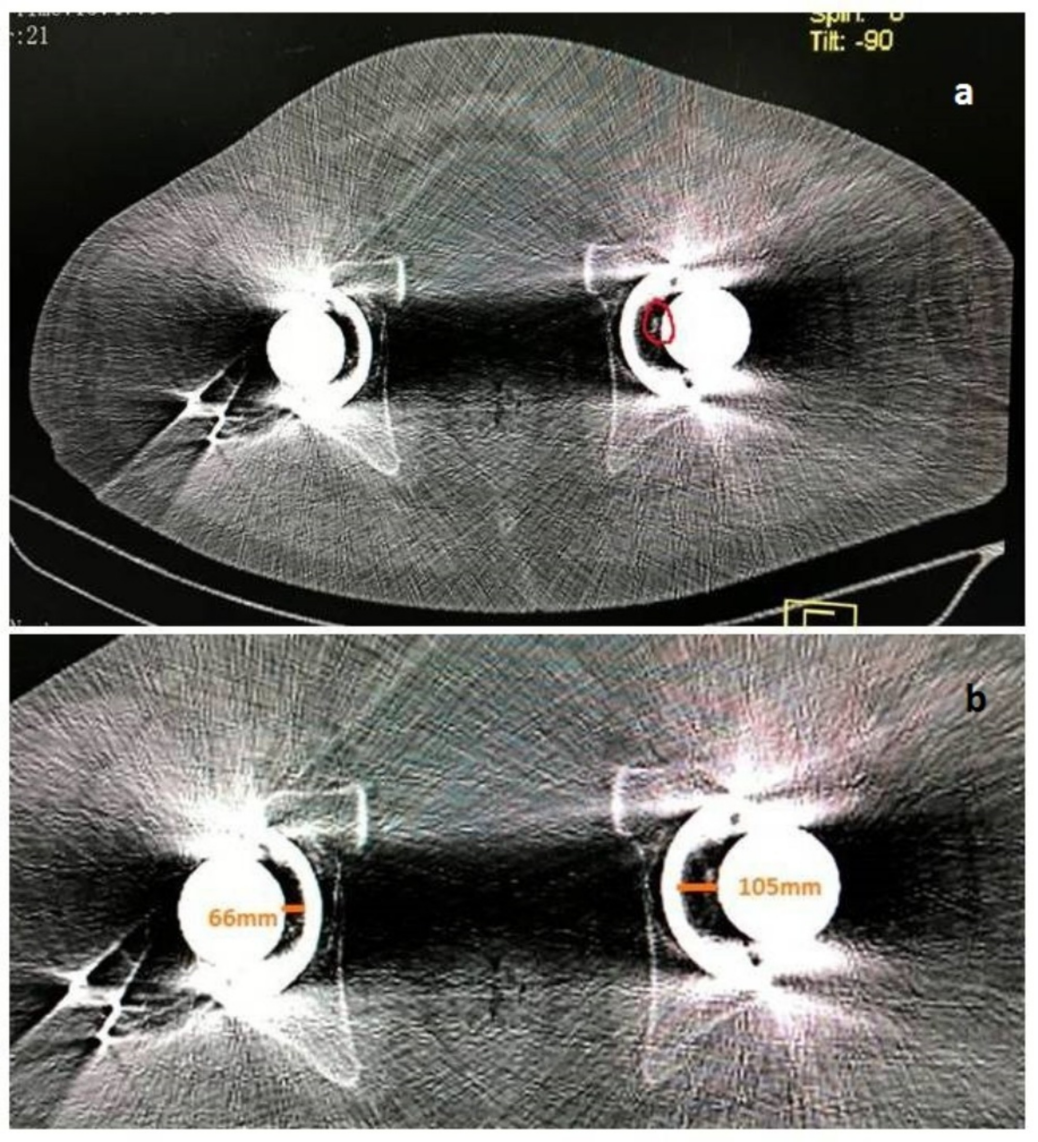
Axial section on computed tomography. a) The loose bone piece (red encirclage). b) The distance from the center of the acetabular shell to that of the femoral head is also increased.

We undertook an urgent computed tomography (CT) to identify the cause, presumed to be an intra-articular obstacle. The axial cuts of CT showed a piece of bone inside the joint that leads to an increase in joint space (Figures 1b, 2a, 2b).

**Figure 3:**
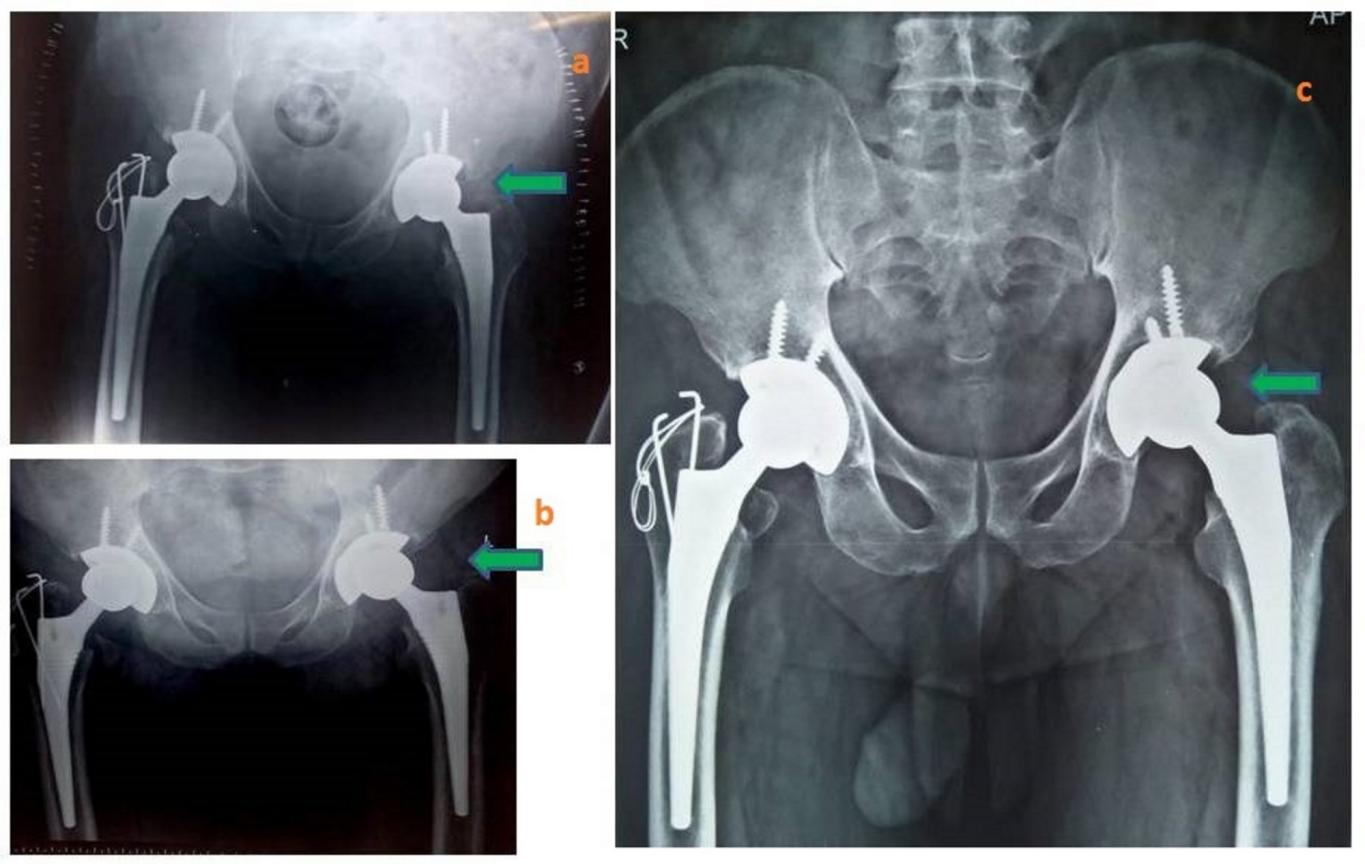
Postoperative and follow-up radiograph. a) Immediate postoperative imaging after re-exploration showing a bilateral congruent reduction. b) Follow-up X-ray showing the same alignment at three months. c) Follow-up X-ray at 18 months.

The patient was taken up for surgical re-exploration, and the obstructive bone piece (an osteophyte) was removed. A fluoroscopy then confirmed concentric reduction (Figure 3a).

Postoperatively, the patient followed a non-weight-bearing protocol for three weeks, followed by gradual mobilization with walker. At the 1.5-year follow-up, the patient’s Harris hip score is 91 and findings on X-ray are satisfactory (Figure 3c).

## Discussion

Total hip replacement has been rightly hailed as the “operation of the century” because of satisfactory outcomes in so numerous patients [1]. Dislocation is a well-known complication following THA, and is more common in revision THA as compared to a primary THA [2]. The present complication is an unexpected result and that was not observed intraoperatively. The acetabulum was cleared of all debris after a through lavage and the head was reduced with traction under direct vision. Post-operatively, a bedside X-ray showed the head was proud with non-centric reduction on the left side. This observation was facilitated by the fact that the X-ray displayed both sides with the same implant that was placed in a single sitting that eased a comparison. There remains a possibility that this occurrence could have been missed if only one side was being operated. A number of factors could be responsible for such a consequence in the immediate postoperative period. This complication is the result of a slipping/non-locking of the acetabular liner leading to disassociation [4]. A variety of intra-articular foreign bodies, such as gauze piece or bone cement (cemented THA), broken/loose polythene, or ceramic liner have also been reported to cause non-concentric reduction [5-7]. Broken liners become entrapped during reduction of a traumatic dislocation following a THA. Chan et al. reported a case of sciatic nerve entrapment during reduction [8]. However, they diagnosed this complication based on clinical findings, as radiological signs were absent. In the present case, a small piece of bone had lodged itself in the articular surface during the reduction of the final implants. We hypothesize that an innocuous supero-lateral osteophyte might have broken off during the reduction maneuver as the head was pulled toward the acetabulum. The literature suggests that such osteophytes may be absorbed in due course and can be ignored [9]. This report highlights the importance that all osteophytes should be removed; they should be followed by an extensive wash and suction to clear small bony/cement debris and, most importantly, the reduction should be undertaken under direct vision to prevent such a complication.

## Conclusions

A benign supero-lateral osteophyte that is useless and relatively harmless can break and become entrapped during a reduction maneuver in a THA. This is undesirable, and young surgeons should be aware of such a possibility. In case of any suspicion of a non-concentric reduction, CT is beneficial for diagnosis.
